# Characterization of a novel endothelial biosensor assay reveals increased cumulative serum inflammatory potential in stabilized coronary artery disease patients

**DOI:** 10.1186/s12967-015-0457-5

**Published:** 2015-03-22

**Authors:** Heidi Cung, Mario J Aragon, Katherine Zychowski, Joe R Anderson, James Nawarskas, Carlos Roldan, Akshay Sood, Clifford Qualls, Matthew J Campen

**Affiliations:** Department of Pharmaceutical Sciences, College of Pharmacy, University of New Mexico, Albuquerque, NM USA; Department of Pharmacy Practice and Administrative Sciences, College of Pharmacy, University of New Mexico, Albuquerque, NM USA; Department of Internal Medicine, University of New Mexico, Albuquerque, NM USA; Department of Biostatistics, School of Medicine, University of New Mexico, Albuquerque, NM USA

**Keywords:** Coronary artery disease, Biomarker, Inflammatory, Serum, Endothelial

## Abstract

**Background:**

Vascular disease is promoted by systemic inflammation that can arise from sites distal to the affected vessels. We sought to characterize the net inflammatory potential of serum from patients with coronary artery disease (CAD) using cultured endothelial cells as a cumulative biosensor.

**Methods and results:**

Serum samples from CAD patients (N = 45) and healthy control subjects (N = 48) were incubated with primary human coronary artery endothelial cells at a 1:10 dilution for 4 h, followed by isolation of the cellular RNA. Alteration of inflammation-responsive elements (adhesion molecules and cytokines) was assessed by gene expression. Specific indicators included intercellular adhesion molecule-1 (ICAM-1), vascular cell adhesion molecule-1 (VCAM-1), and interleukin-8 (IL-8). Additionally, the cytokine levels in serum samples from all subjects were quantified. Serum from CAD subjects induced greater endothelial ICAM-1, VCAM-1, and IL-8 expression compared to healthy control serum (p < 0.001 for each analysis). The three indicators of inflammatory potential (ICAM-1, VCAM-1, and IL-8 mRNA) trended independently of each other and also of serum inflammatory biomarkers. IL-8 expression correlated negatively with serum HDL levels but positively correlated with VLDL, plasminogen activator inhibitor-1 and C-reactive protein. Interestingly, serum levels of cytokines in CAD patients were not statistically different from healthy control subjects. A year of follow-up in a sub-group of CAD subjects revealed relatively stable measures.

**Conclusions:**

As yet unidentified circulating factors in the serum of CAD patients appear to activate endothelial cells, leading to upregulation of adhesion molecules and chemokines. This cumulative assay performed well in terms of discriminating patients with CAD compared to healthy subjects, with greater range and specificity than specific inflammatory markers.

## Introduction

Vascular disease is an inflammatory condition wherein activated endothelial cells mediate the recruitment of immune cells into damaged areas of the vessel wall, promoting oxidative injury, pathological lesion growth and increased histological complexity [1]. Systemic inflammation arising at distal sites, such as the lungs or liver, may also contribute to the inflamed state of coronary or cerebral vessels through unknown mechanisms [2-6]. Circulating factors likely have a causal role in promoting endothelial cell activation, a state characterized by presentation of adhesion molecules and release of chemokines [7,8]. Specific circulating acute phase proteins such as C-Reactive Protein (CRP) and tumor necrosis factor-α (TNFα) are both associated with vascular disease and can independently activate endothelial cells via NF-κB pathways [9-11]. However, because the blood contains thousands of factors, the discrete assessment of specific cytokines may fail to capture the overall inflammatory influence that the blood conveys to the endothelial wall. Not surprisingly, such individual factors contribute only modestly to cardiovascular risk prediction or reclassification beyond conventional Framingham risk factors [12] and we propose that this gap between biomarkers and outcome is blurred by the complex composition of the blood and numerous unaccounted factors.

We have developed a novel, less biased approach to assess what we believe to be a functional index of the total inflammatory potential of the serum [13]. By assessing canonical inflammatory response patterns in primary human coronary artery endothelial cells (hCAECs) treated with dilute samples of serum or plasma, the endothelial cells act as biosensors of the complete serum milieu. This assay was initially developed to explore the relatively modest impact of inhaled pollutants on cardiovascular health. It was found that serum from healthy individuals, when obtained shortly following controlled exposures to diesel engine emissions or nitrogen dioxide, carried a greater potential for inducing endothelial cell adhesion molecules and chemokines compared to sham exposure [13]. Similarly, whole serum has been used to identify a net inflammatory influence of cigarette smoking, leading to reductions of eNOS expression and increased reactive oxygen species [14,15]. Conversely, in a larger cohort of healthy subjects taking a month-long regimen of a resveratrol-containing nutraceutical, we found that circulating inflammatory potential decreased relative to pre-treatment plasma, while placebo administration caused no clear trend and no changes in other biometrics were observed [16]. The overall approach has also proven useful in animal toxicology research [17,18], but the linkage to clinically-relevant disease has yet to be assessed.

Thus, the value of this assay paradigm remains uncertain with respect to clinical outcomes. Many questions remain unanswered, such as whether elevations in net serum inflammatory potential contribute to chronic cardiovascular disease and whether such measures have predictive or diagnostic value. The present study conducted a side-by-side comparison of stabilized coronary artery disease patients on standard-of-care medication with a separate cohort of healthy subjects.

## Methods

### Patient population

Patients with CAD were recruited while hospitalized at the University of New Mexico Hospital for an acute coronary syndrome event (unstable angina, non-ST elevation myocardial infarction, or ST-elevation myocardial infarction) to participate in a health outcomes study designed to assess the benefit of an interdisciplinary cardiovascular risk reduction clinic (CRRC), as compared to usual care. The study was approved by the University of New Mexico Health Sciences Center Human Research Review Committee and all study patients provided informed consent.

As part of the study, blood samples were collected from all patients for assessment of a variety of traditional and non-traditional risk factors (lipids, HbA1c, homocysteine, malondialdehyde [MDA], plasminogen activator inhibitor-1) and a portion of the samples were stored for future use. Patients randomized to the CRRC were evaluated by both a cardiologist and a clinical pharmacist, who devised an appropriate treatment plan based upon identified cardiovascular disease risk factors. Patients randomized to usual care received follow-up at the discretion of their primary care providers and/or specialist providers.

The majority of CAD patients were on standard-of-care pharmacotherapy, including strategies to reduce cholesterol, glucose, blood pressure, platelet aggregation, and tobacco cessation where appropriate. The serum samples used in this analysis were collected at the initial outpatient follow-up study visit to the CRRC.

Healthy control subjects were recruited through the University of New Mexico Clinical and Translational Science Center for routine health screens. Subjects were recruited by newspaper or radio advertisements from the community. Exclusion criteria included - 1) history of diabetes mellitus, atherosclerotic cardiovascular disease, chronic kidney disease, or anorexia nervosa; 2) use of statin class of drugs; 3) current smoking or having quit smoking within previous two months; 4) pregnancy and nursing state; 5) presence of lung diseases other than asthma; 6) stroke in prior 3 months; 7) aortic aneurysm; and 8) failure to expectorate adequate-quality sputum in response to induction. In addition, all tests were delayed in the event of an acute infection or surgery within the prior 4 weeks and respiratory tract infections or asthma exacerbations within the prior 8 weeks to minimize the effect of these factors on measurements. For actively menstruating women, the testing was done within 3–14 days following the cessation of menstrual flow (a period of high estrogen and low progesterone) to standardize the effect of sex hormones.

### Bioelectrical impedance

CAD participants were assessed for body composition in a fasting state using a Quantum II Bioelectrical Impedance Analyzer (RJL Systems, Clinton Township, MI) using the method described by Heyward [19]. Results for bioelectric impedance were analyzed with Comprehensive Body Composition Software (Human Kinetics, 1997, Champaign, Ill) and percent body fat mass was calculated using standard reference equations appropriate for race and gender [19].

### Cell culture assays

**Figure 1:**
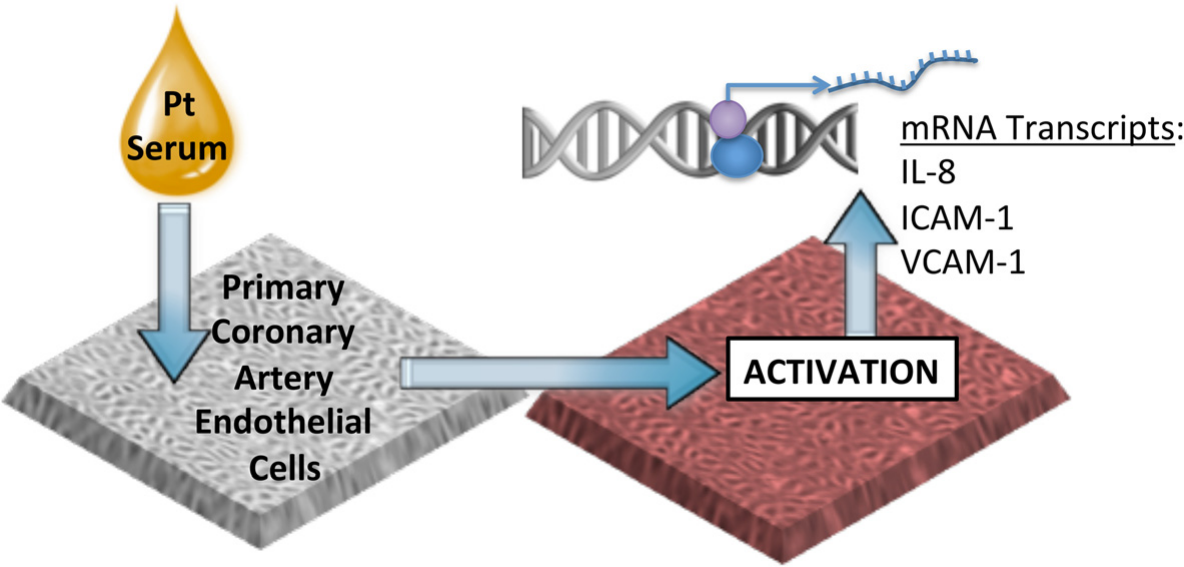
**Endothelial cell biosensor approach.** Primary coronary artery endothelial cells are incubated with 10% serum from individual patients for 4 h, followed by isolation of RNA, and then assayed for transcriptional responses.

The endothelial cell biosensor assay (Figure 1) was conducted as previously described [13,16]. Briefly, hCAECs (Lonza, Allendale, NJ) were seeded in a 24-well plate and grown to confluence in complete media (Lonza). The cells were then serum-starved with Basal Media (Lonza) for 24 hours prior to exposure. Confluent hCAEC were incubated with 10% serum obtained from coronary artery disease (CAD) patients (ie., 50 μl serum in 450 μl media, per well) or 10% serum obtained from otherwise healthy individuals. Each plate of cells was treated with approximately equal numbers of subjects from each group in a randomized, blinded fashion. The samples were incubated for 4 hours at 37°C. RNA was extracted from the cells and cDNA was made using a ThermoCycler (Model # PTC-200; MJ Research). The cDNA was used to determine gene expression via quantitative polymerase chain reaction (LightCycler 480 II, Roche, Indianapolis, IN). Specific targets included interleukin-8 (IL-8), intercellular adhesion molecule-1 (ICAM-1), and vascular cell adhesion molecule-1 (VCAM-1), with TATA-box protein used as a housekeeping gene.

### Serum cytokine measurements

Serum samples from healthy and CAD patients were analyzed using an electrochemiluminescence detection system (Meso Scale Discovery, Rockville, MD). The serum samples were analyzed for the presence of CRP, soluble ICAM-1, soluble VCAM-1, serum amyloid A (SAA), TNF-α, IL-6, IL-8, and IL-1β using commercially-available kits.

### Data analysis

Gene expression data were log_10_-transformed for normality. Data were analyzed between groups (CAD versus control) via Satterthwaite’s t-test (SAS v9.4). A multivariate analysis confirmed this difference between CAD and control outcomes after adjusting for age, sex, and BMI. Correlational (Spearman) and multivariate analysis was carried out on the CAD patient outcomes relative to demographic and clinical data (SAS).

## Results

### Cohort data

A total of 48 patients with CAD and 45 healthy control subjects were included in the study. Subjects were self-selected and inclusion criteria allowed for a wide range of CAD etiology and manifestations. Table 1 summarizes the demographic information. The CAD patients in this unmatched study were expectedly older than control subjects and had a disproportionate prevalence of CV risk factors, including diabetes, body mass index (BMI), hypertension, hypercholesterolemia and current tobacco use. Importantly, a sufficiently overlapping range of demographic factors permitted multivariate assessment to eliminate all potential major effect modifiers, in terms of endothelial cell responses to serum, described below. The CAD cohort was comprised of patients with single and multiple vessel disease, and were recorded on admission as being a mix of non-ST segment elevation myocardial infarction (NSTEMI) and ST segment elevation myocardial infarction (STEMI), as well as unstable angina. Table 2 provides all additional personal health data from the CAD patient population that were available.

**Table 1:** **Basic demographics for the healthy and coronary artery disease subjects**

	**Healthy controls (N = 45)**	**Coronary artery disease patients (n = 48)**
**Age (Mean ± SE; range)**	31.7 ± 13.5; 18-61	56.5 ± 7.9; 36-77
**Gender (% female)**	71%	41.7%
**BMI (Mean ± SE; range)**	22.3 ± 1.7; 18.7-25.5	29.9 ± 6.4; 17.2-48.2
**Fat composition (Bioelectric impedence)**	25.27 ± 6.5; 12.0-40.0	34.3 ± 10.5; 18.5-56.3
**Race**	9.3% Native American/Alaskan	57.9% Hispanic
	4.7% Asian	36.8% White
	9.3% African	5.3% Other
	76.7% Caucasian (29% Hispanic)	
**Comorbidities**		
**Diabetes**	0%	58.3%
**Hypertension**	0%	22.9%
**Smoking**	0%	50%
**Extent of CAD (single, multi vessel), N (%)**	NA	20 (42%), 28 (58%)
**Reason for admission (NSTEMI, STEMI, unstable Angina), N (%)**	NA	14 (29%), 21 (44%), 13 (27%)

**Table 2:** **Further clinical data from CAD cohort**

	**Mean**	**SD**	**Min**	**Max**
**Total cholesterol (mg/dl)**	149.9	39.4	76	241
**LDL**	88.6	31.7	34	175
**HDL**	40.2	12.1	25	88
**HDL2**	9.3	4.9	4	32
**HDL3**	30.9	7.9	18	56
**VLDL**	22.0	8.3	12	64
**VLDL 1,2**	9.8	3.2	5.6	20
**VLDL 3**	18.1	46.2	7	341
**Triglycerides**	156.1	74.8	5	323
**Lp(a)**	6.8	3.9	3	17
**IDL**	8.4	5.5	1	24
**Glucose (mg/dL)**	115.8	37.5	81	265
**HbA1c(%)**	7.63	1.88	5.4	11.4
**Homocysteine**	9.25	4.00	4.6	24.6
**Insulin (uIU/mL)**	14.09	8.02	2.4	43.5
**Fibrinogen (mg/dL)**	363.2	89.8	231	660
**PAI (IU/ml)**	15.1	13.1	0.1	50
**UrMicAlb (mgALB/gCrea)**	77.1	271.6	0.6	1481.6
**SBP**	130	19	100	186
**DBP**	74	11	48	100
**HR**	67	11	43	89
**Weight(kg)**	85.5	17.4	53.4	123.25
**Bioelectric impedence**	36.1	9.08	22.6	48.8
**MDA**	0.236	0.089	0.094	0.414
**Days post-event**	400	568	16	2748
**Medication data**				
**Statin use**	75%			
**Antiplatelet use**	97.9%			
**Glucose control**	25%			
**ACE inhibitors or ARBs**	75%			
**β-blockers**	68.8%			

### Serum from CAD patients has greater inflammatory potential than serum from healthy subjects

**Figure 2:**
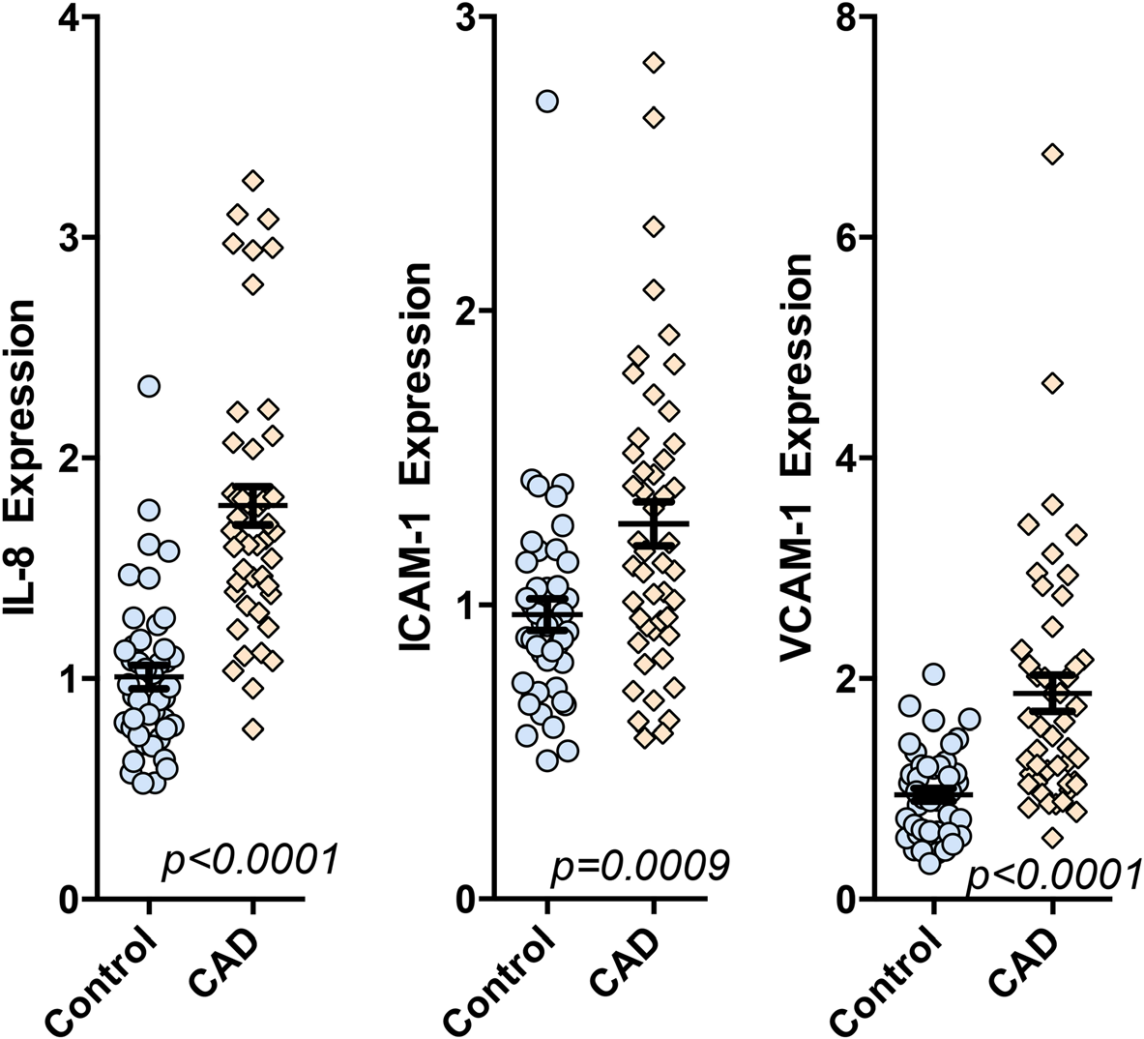
**Endothelial cell expression of IL-8, ICAM-1, and VCAM-1 mRNA in response to incubation with 10% serum from healthy controls or CAD cohort.** Significance was assessed with a Student’s t-test. The differences between groups remained significant after controlling for age, sex, and body mass index, and all potential interactions using a multivariate analysis of variance (general linear model).

hCAECs treated with 10% serum from CAD subjects showed substantial increases in IL-8, ICAM-1, and VCAM-1 expression, compared to hCAECs treated with serum from healthy control subjects (Figure 2). Endothelial mRNA expression of IL-8 and VCAM-1 were approximately 80% higher in hCAECs treated with CAD patient serum compared to controls (p < 0.0001). ICAM-1 expression was more stable at approximately 30% above control (P = 0.0009) and with less overall variability. The distribution patterns of gene expression for each of these markers were notably different and correlations among the marker expression from individuals varied considerably. Cross-correlations ranged from moderate (R = 0.534 for VCAM-1 and ICAM-1) to low (R = 0.201 for ICAM-1 and IL-8), implying that these responses might be influenced by different factors – or cumulative factors - in the serum.

### Relationship of endothelial responses to serum to time post-event and longitudinal trends

**Figure 3:**
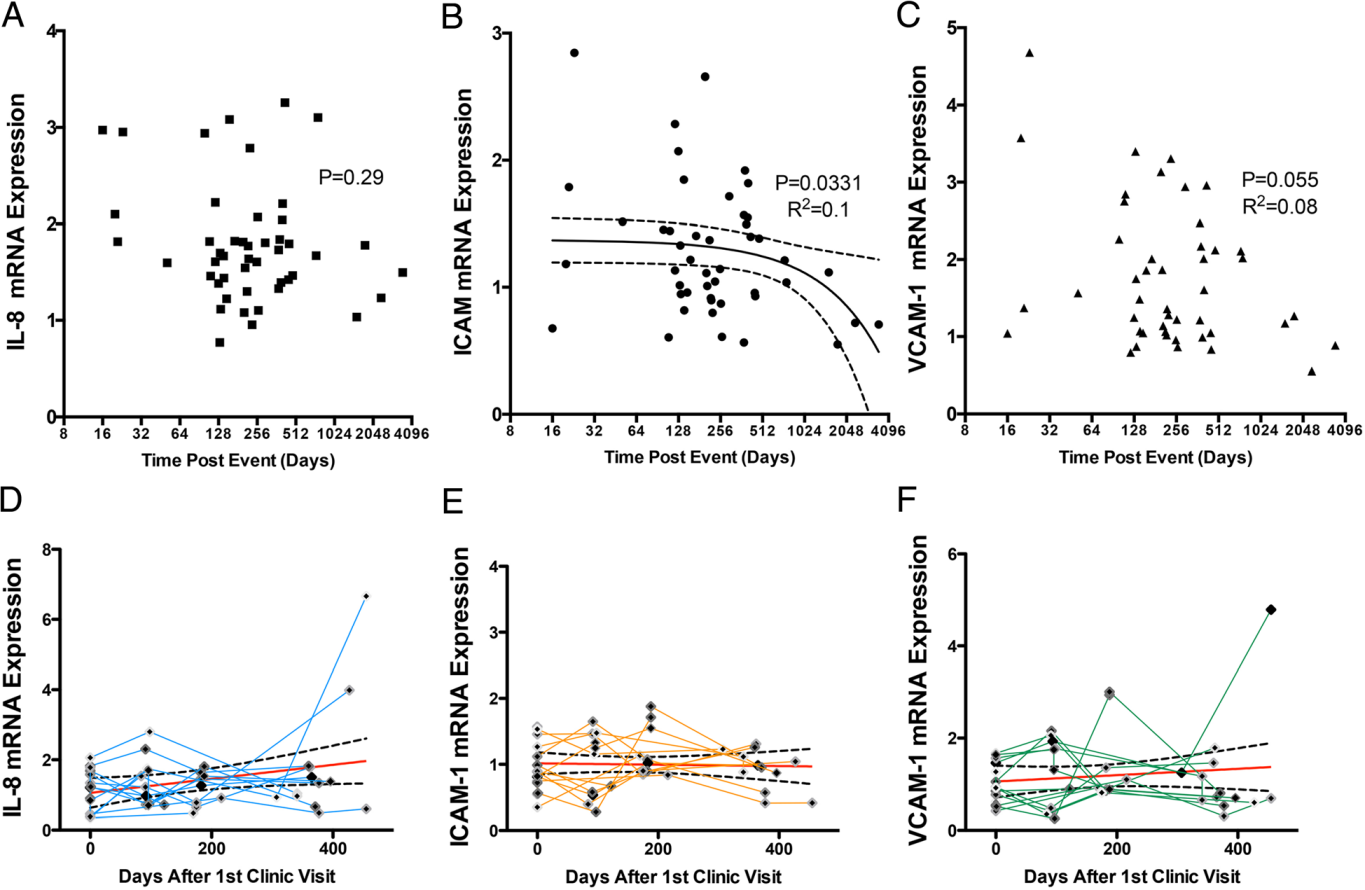
**Temporal trends on serum inflammatory potential.** Cross-sectional samples were obtained from 16d to several years after initial cardiac events **(A-C)**. The time post-event had minimal effect on IL8 mRNA expression, but factors in the serum that drove ICAM-1 and VCAM-1 expression may have been higher acutely following the event. Longitudinal serum samples obtained approximately every 3 months from a limited cohort of CAD subjects were assessed for inflammatory potential independently from healthy subjects **(D-F)**.

As many serum components can be acutely altered as a result of major cardiac events, or may trend with age and/or time, we examined the impact of temporality on serum-treated hCAEC mRNA expression in both the cross-sectional samples, as well as with a sub-cohort of longitudinally followed CAD subjects. All samples were obtained from patients with known CAD, either from having previously suffered an acute myocardial infarction or angina attack. The average time post-event was slightly greater than one year (400 days), but 5 subjects enrolled in the study within 2 months of the precipitating event and 6 subjects were greater than 2 years past their event. The earliest enrolled subject had an event 16 days prior to the initial visit. In general, this cross-sectional data set did not reveal strong trends of temporal resolution of the bioactivity of the serum (Figure 3A-C). For serum-treated hCAEC IL-8 expression, there were no significant trends. hCAEC mRNA for ICAM-1 and VCAM-1 demonstrated modest reductions relative to the time post-event (P = 0.033 and 0.055, R^2^ = 0.10 and 0.08, respectively). As serum samples more proximal to the event than 16 days were not available, it is not known if the serum obtained within hours or days after the event might be more inflammatory. However, from months-to-years after an event, the data suggest considerable stability.

In a limited cohort of subjects (N = 11), four longitudinally obtained serum samples were available for approximately one year after the initial clinic visit (Figure 3D-F). We examined the relative serum inflammatory potential trends, in terms of activation of hCAEC responses, in these subjects and found that no absolute trends were observed over the year of follow-up. However, clear variability in endothelial cell responses to serum obtained at different visits was evident. As the medical records for these subjects lacked temporal details in terms of symptoms and medications, we are unable to ascribe such short-term trends to any clear change in health status. Importantly, the lack of long term trends in this sub-cohort of CAD subjects on standard of care medication is consistent with the fact that all of these subjects survived beyond this year of follow-up.

### CAD Manifestation effects on serum inflammatory potential

**Figure 4:**
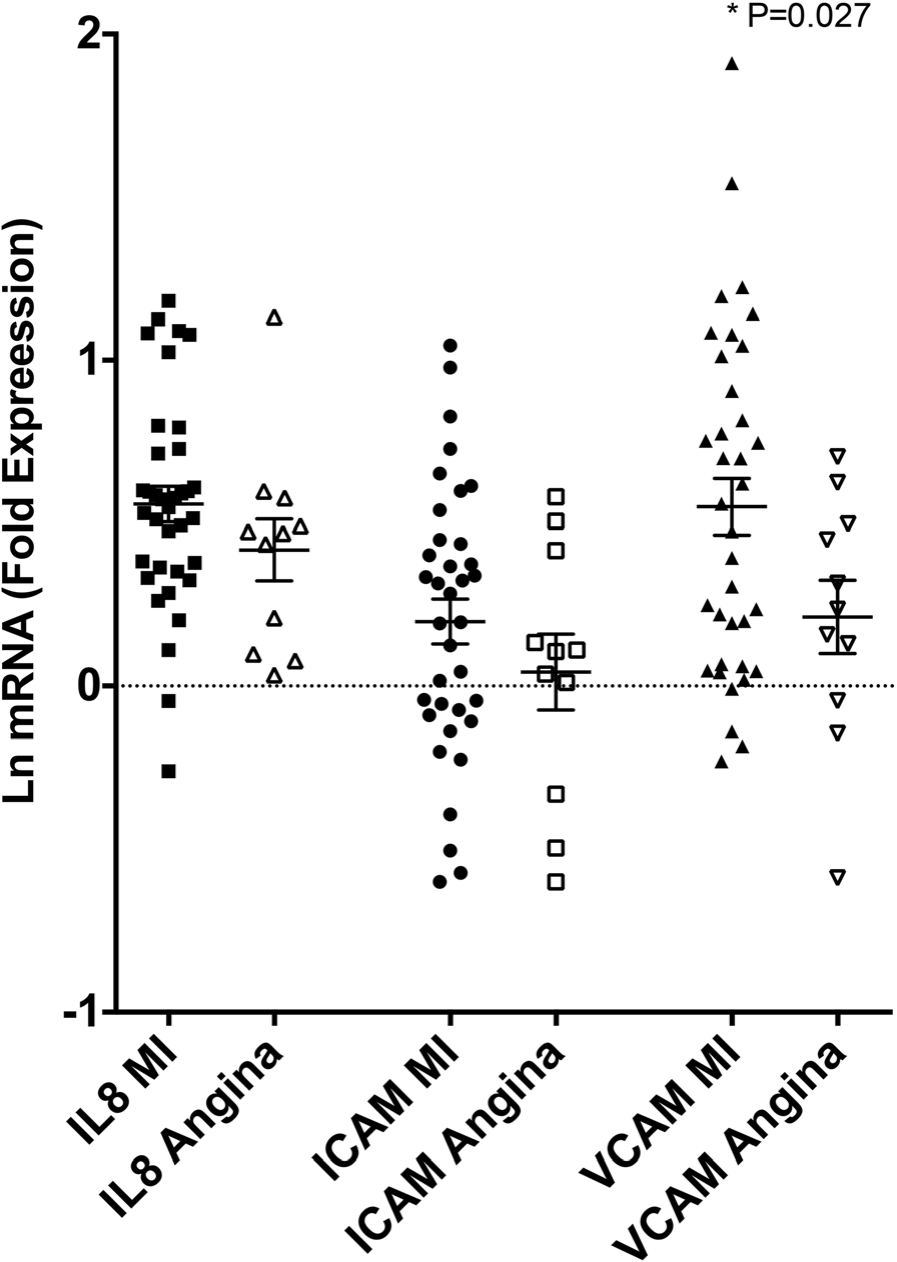
**Serum from subjects with diagnosed myocardial infarction (MI) induced greater VCAM-1 induction compared to serum from subjects with unstable angina (P = 0.027 by one-tailed t-test on natural-log-transformed data).** Both IL-8 and ICAM-1 trends were non-significant.

Medical records allowed for initial event categorization of CAD subjects. We therefore considered an intra-cohort comparison for subjects whose initial presentation was a myocardial infarction (with or without ST elevation) or unstable angina (Figure 4). A slight trend for increased expression of the gene transcripts was noted for patients admitting with an MI, which was only significant for VCAM-1 (P = 0.0266). No significant differences were observed between subjects admitting with ST-elevation MI or non-ST elevation MI (data not shown). These findings are consistent with the hypothesis that greater inflammatory potential in the serum may lead to more severe vascular pathology, although the results are associative.

### Endothelial cell responses to serum were not impacted by sex, age or BMI

**Figure 5:**
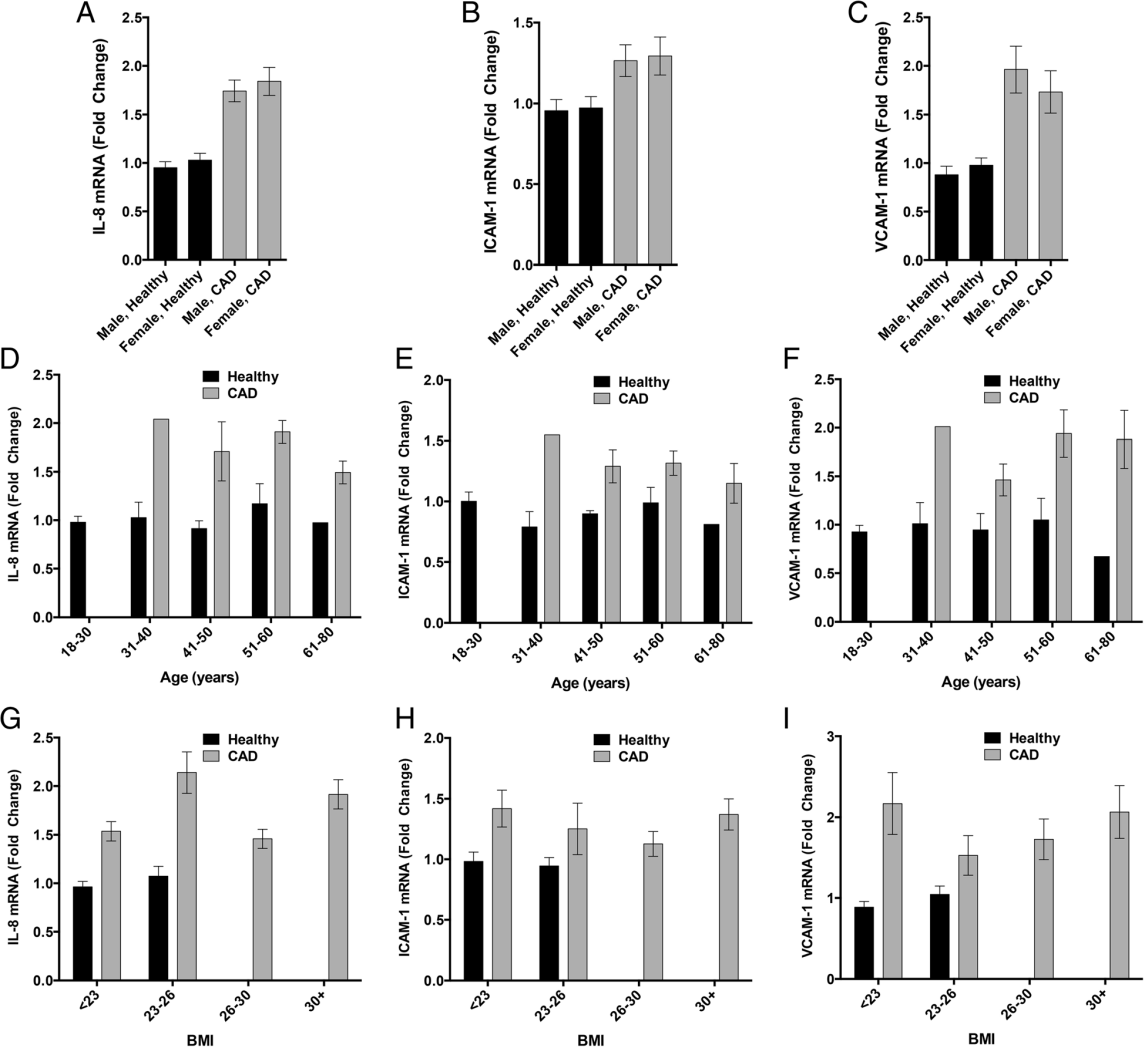
**Potential effect modifiers sex, Age, and BMI have no clear influence on serum inflammatory potential.** Sex related differences are negligible in determining the differences between healthy and CAD serum-induced endothelial cell responses **(A-C)**. Similarly, Age does not appear to drive any trends for serum-induced IL-8, ICAM-1, or VCAM-1 mRNA expression **(D-F)**. While BMI is limited in the control population, the lack of consistent trends in controls and CAD across various BMI ranges reduces confidence that this factor has a clear role in drive the serum inflammatory potential **(G-I)**.

Importantly, the significant differences in endothelial expression of IL-8, ICAM-1, and VCAM-1 in response to patient serum remained significant even after adjusting for the potential major effect modifiers of sex, age, and BMI that were clearly different between cohorts. Direct comparisons across sex, age, and BMI are shown in Figure 5. For each mRNA target, it is clear that CAD subjects were elevated compared to healthy controls, but there was not impact of sex on the endothelial cell responses to serum in either healthy or CAD cohorts (Figure 5A-C). Similarly, age and BMI showed no clear trends across the complete range of either variable (Figure 5D-I).

Multivariate regression was also applied to test the transcriptional outcomes relative to the potential effect modifiers. In considering all variables and potential interactions among sex, age, and BMI as confounders, differences between healthy and CAD subjects for expression of IL-8, ICAM-1, and VCAM-1 remained significant. Thus, despite the unmatched cohorts, there is high confidence that the CAD condition alone imparts an independent pro-inflammatory effect on the serum.

### Demographic factors that correlate with hCAEC responses

In exploring potential factors that may contribute to serum-induced endothelial cell responses, we found a number of demographic factors in CAD patients that correlated with IL-8, ICAM-1 and VCAM-1 expression (Table 3). IL-8 expression associated with a number of factors, positively trending with serum levels of VLDL1/2, CRP, and PAI-1, while negatively trending with serum glucose and HDL levels. VCAM-1 expression correlated with both smoking and serum homocysteine levels, while ICAM-1 expression correlated with age, serum homocysteine, and MDA. While serum homocysteine trended negatively with inflammatory potential, it should be noted that out of 51 patients, only 3 values for homocysteine were out of normal range, and only two of these were paired with a measure for VCAM-1 mRNA. Fewer demographic and personal health data were available for our control population, and those few factors analyzed (age, BMI, gender) did not correlate with endothelial cell responses to serum.

**Table 3:** **Factors in CAD patients that correlate with hCAECs responses to serum**

**IL-8**	***R***	***p-value***
Diabetic	0.262	0.079
Glucose	−0.358	0.013
HDL	−0.298	0.039
HDL2	−0.272	0.061
HDL3	−0.283	0.052
VLDL1/2	0.254	0.081
C-Reactive protein	0.329	0.038
Plasminogen activator inhibitor-1	0.367	0.077
VCAM-1	*R*	*p-value*
Smoking	0.263	0.074
Homocysteine	−0.274	0.059
ICAM-1	*R*	*p-value*
Age	−0.301	0.038
Homocysteine	−0.368	0.010
Time post-event	−0.312	0.033

As IL-8 expression had the most robust correlates, we developed a regression model to better understand the factors that predict this outcome (Table 4). This model confirmed the outcomes in the correlational analysis, providing confidence for the roles of serum VLDL, CRP, and glucose levels in driving the IL-8 related inflammatory potential. However, these variables only explained a small portion of the inflammatory potential, indicating that other unmeasured factors are also mediators.

**Table 4:** **Predictive variables for IL8 response using a linear regression model**

**Variable**	**Parameter estimate**	**Std error**	**t value**	**Pr > | t |**	**Standardized estimate**
Intercept	0.141	0.0815	1.73	0.092	0
VLDL 1/2	0.0199	0.0062	3.22	0.0027	0.438
CRP	0.0100	0.0028	3.54	0.0011	0.474
Glucose	−0.0012	0.0005	−2.22	0.0328	−0.298

### Cytokine Levels did not explain the difference in serum inflammatory potential between groups

**Figure 6:**
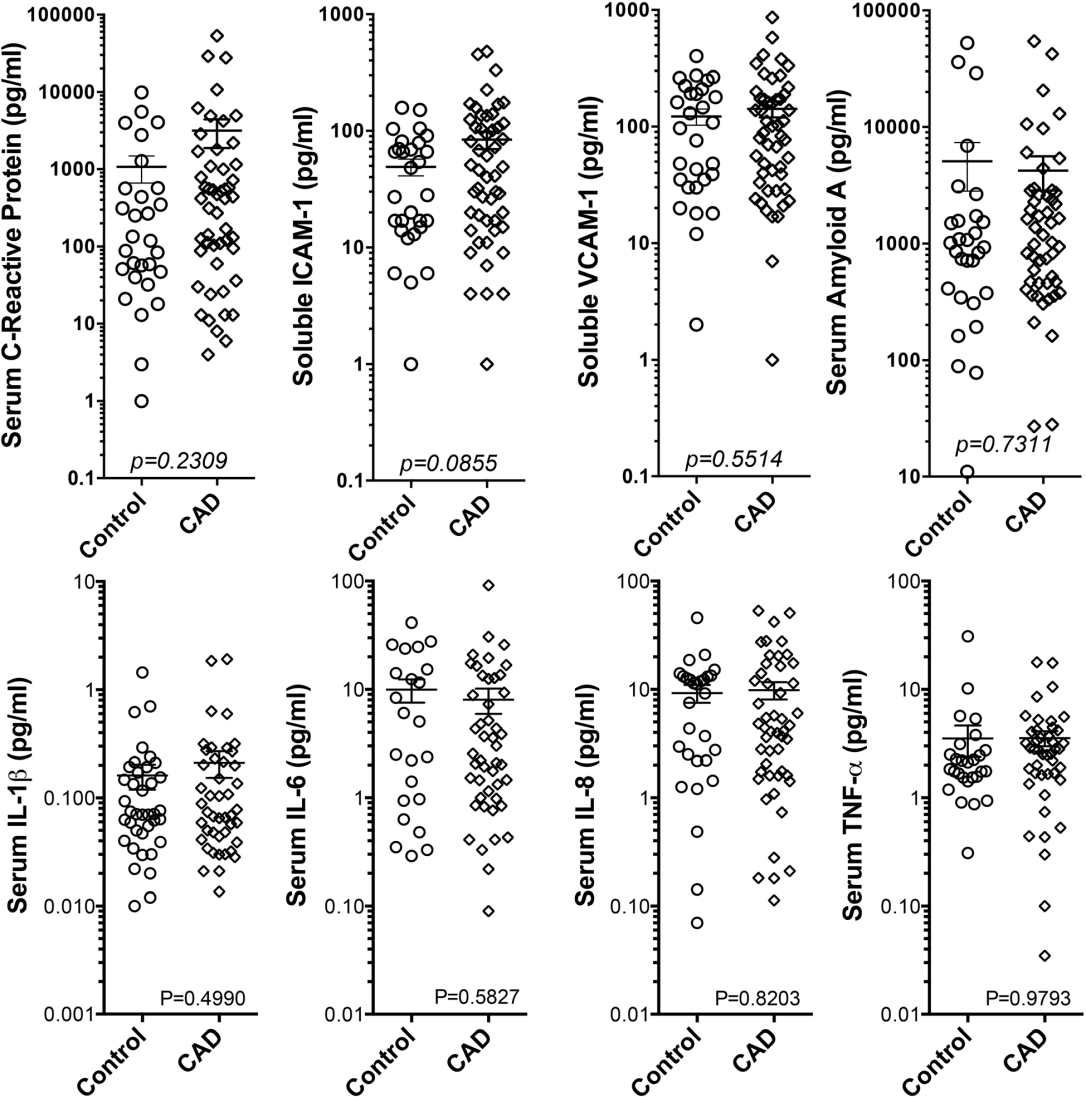
**Cytokine and acute phase proteins measured by electrochemiluminescence in serum from healthy controls and CAD cohort.** Significance was assessed with a Student’s t-test.

To assess whether inflammatory cytokines might explain differences in the serum inflammatory potential between healthy and CAD subjects, we measured 8 key inflammatory cytokines in serum from all patients using an electrochemiluminescence technique (Figure 6). No statistically significant differences were noted for IL-1β, IL-6, IL-8, TNFα, C-Reactive Protein, soluble VCAM-1, or Serum Amyloid A, either in terms of mean or distribution, between healthy and CAD cohorts. A modest, but non-significant increase in soluble ICAM-1 was noted however (49.1 ± 8.1 controls versus 84.1 ± 14.4 in CAD patients, P = 0.086).

## Discussion

The present study assessed cumulative serum inflammatory potential from a cohort of CAD patients in stable condition under standard-of-care for medications and lifestyle adjustments, using an endothelial biosensor assay. Utilization of circulating biomarkers has proven valuable for diagnostic and prognostic purposes in atherosclerotic disease, but even the most predictive single mediators provide only small incremental improvements in overall prediction of risk or patient reclassification [20,21]. Endothelial inflammatory response to serum from CAD patients was significantly increased compared to responses to control subject serum, despite observing that most measured circulating cytokines were no different between these cohorts. Additionally, our characterization of the temporal dynamics of this assay reveals stability over the course of a year of follow-up, albeit with some lability, reflecting a generally successful course of treatment. The value of this assay paradigm - using endothelial cells as “biosensors” of circulating inflammatory potential - remains incompletely appreciated but highly promising, as the ability to discriminate clinical populations in a manner consistent with a diagnosed clinical condition would be a clear advantage over assessment of single or even multiple biomarkers that can not discriminate from healthy subjects.

Serum obtained from patients with a previous history of a major coronary vascular event showed a greater potential for inducing endothelial cell inflammatory responses, such as IL-8, VCAM-1, and ICAM-1 expression, compared to serum from healthy individuals. The CAD patients, all substantially post-event (mean = 1.1y; range = 16d − 7.5y), were expectedly receiving numerous medications (>7) as standard-of-care. CAD subjects were generally prescribed at least one lipid control agent (*e.g*., statin), an antiplatelet agent (*e.g*., aspirin and/or clopidogrel), and patients with Type 2 Diabetes Mellitus were also taking at least one glucose control agent (Table 2). Many of these medications have reported pleiotropic anti-inflammatory effects, which may explain the reduction of cytokine levels to control cohort values. In recent reports, statins have been shown to reduce CRP levels. For instance 6 months of 10 mg/day of Atorvastatin led to a 26% drop in CRP and also diminished the predictive value of CRP, in terms of risk for major adverse cardiovascular events [22]. Simvastatin therapy provides similar results, while combined simvastatin and ezetimibe led to a >35% reduction on CRP [23]. Other standard of care medications likely contribute further to this CRP lowering effect, including aspirin [24], clopidogrel [25], and glucose control medications such as rosiglitazone [26]. Other inflammatory cytokines, including IL-6 and TNF-α, appear to be similarly affected by statins and antiplatelet medications [27,28]. Thus, the ability to differentiate healthy control subjects and patients with diagnosed CAD using such cytokine markers is substantially hampered once such medications are implemented [22]. Importantly, serum inflammatory potential determined with the novel endothelial bioassay remained elevated compared to healthy control subjects, despite the medication use, although the prognostic/diagnostic value of such outcomes remains to be defined.

In previous research, we have found this endothelial bioassay paradigm valuable for assessing potential benefits of grape seed extract with added resveratrol in a healthy adult population [16]. Whereas serum markers of inflammation, such as interferon-gamma and interleukin-1β, did not change with resveratrol/grape seed extract treatment, compared to placebo, endothelial cells incubated with serum obtained after the month-long resveratrol/grape seed extract regimen exhibited consistently lower expression of IL-8, ICAM-1, and VCAM-1 mRNA compared to baseline responses. Conversely, in studies of air pollution health effects in another healthy cohort, we observed an increase in the serum inflammatory potential following inhalation of diesel exhaust emissions and nitrogen dioxide [13]. The degree of inflammatory potential change was smaller than that seen with the differences in the present study, on the order of 20-30% increases, acutely, compared to 30-90% increases for mRNA responses to serum between CAD patients and a healthy cohort. These outcomes are consistent with earlier research using this general approach. Barbieri and colleagues found that serum from cigarette smokers could induce COX-2 and intracellular reactive oxygen species in a manner that appeared dependent on TNFα and interleukin-1β [15]. Similarly, Barua and colleagues treated hCAECs with serum from smokers and found a down-regulation of eNOS protein activity that was reversible by the addition of superoxide dismutase and catalase [14]. The present study of CAD provides a vital clinical anchor for understanding these earlier studies that used this serum inflammatory potential assay, showing that elevated endothelial cell responses to serum is consistent with diagnosed vascular disease.

Several study limitations are also viewed as opportunities for future research, as the overall concept of holistic cell-based responses to whole serum remains quite novel and unexplored. For one, while IL-8, VCAM-1, and ICAM-1 mRNA are consistently reported as involved in vascular disease progression, both in clinical studies and basic research, there are a number of important response factors that have not been assessed. It is highly probable that many other transcripts are more responsive to the inflammatory factors in the CAD serum, or that specific proteins may be produced, expressed on the cell surface, or secreted in a selective manner. It is likely that endothelial genomic or proteomic outcome patterns would identify stronger response elements or provide “fingerprint” responses to specific clinical conditions or allow differentiation between acute environmental influences (*i.e.,* diet, air pollution) and chronic syndromes (*i.e.*, type 2 diabetes mellitus or end stage renal disease).

Secondly, we have yet to clearly link endothelial cell responses to serum with outcomes of morbidity and/or mortality, thus the clinical value of the present study remains uncertain. Our previous work suggests that this approach may be valuable for studies of toxicology [13] or therapeutic efficacy [16], and the present study highlights a potential for diagnostic purposes. However, we did not have sufficient subject numbers to conduct a complete follow-up study to determine whether the serum inflammatory potential may help in the prediction of adverse outcomes.

Lastly, there is inherently concern relative to the unmatched study cohorts, despite our findings revealing negligible influence of sex, age, or BMI on the coronary artery endothelial cell transcriptional responses to CAD patient serum. Improved matching in future research will certainly provide greater confidence in this outcome. However, our findings remain notable due to the power of this novel approach to discriminate these populations when conventional inflammatory cytokines did not. There is a vast amount of clinical and research efforts invested in the use of circulating biomarkers. The finding in the present study that CAD patients on standard-of-care medication showed no difference in cytokine profiles from a cohort that was younger, had a lower BMI, and more predominantly female, is naturally a concern and clear motivation for the development of novel diagnostic and prognostic markers, such as pursued in the present study.

## Conclusions

The use of cells as biosensors to detect cumulative circulating inflammatory may have significant clinical value. Serum contains thousands of factors - proteins, lipids, and metabolites - that can be augmented by numerous pathologies. Hundreds of potential drivers of endothelial cell activation and vascular inflammatory pathologies exist, such as myeloperoxidase, oxidized lipids or lipoproteins, advanced glycation endproducts or damage-associate molecular patterns (DAMPs). Additionally, endogenous proteins may be modified (fragmented or adducted) by reactive molecules, leading to altered biological activity or pathological epitopes. The present findings, combined with previous experience with this assay, highlight the potential usefulness of a cumulative bioactivity response, compared to measurements of individual factors to assess an overall inflammatory potential of the circulation. Sophisticated proteomic, lipidomic and metabolomics approaches will naturally be valuable to establish key drivers and link back to clinical course. However, we remain skeptical that any single marker will explain the net inflammatory serum potential; thus, there is need for holistic assessments such as this endothelial biosensor method. Future work is required to address the value of this approach for prediction of risk, efficacy of therapies, or safety of pharmaceuticals.

## Abbreviations

BMI: Body mass index
CRRC: Cardiovascular risk reduction clinic
CAD: Coronary artery disease
CRP: C-reactive protein
HbA1C: Hemoglobin A1C
hCAECs: Human coronary artery endothelial cells
IL-8: Interleukin-8
ICAM-1: Intracellular adhesion molecule-1
MDA: Malondialdehyde
TNFα: Tumor necrosis factor-α
VCAM-1: Vascular cell adhesion molecule-1
